# Multimodal fertility cues in chimpanzees: how body odours complement sexual swellings

**DOI:** 10.1038/s41598-026-70687-z

**Published:** 2026-09-10

**Authors:** Marlen Kücklich, Madita Zetzsche, Sofya Dolotovskaya, Jakob W. Siepmann, Lydia Schmidt, Claudia Wiesner, Brigitte M. Weiß, Anja Widdig

**Affiliations:** 1 Research Group Behavioural Ecology, Institute of Biology, Leipzig University, Talstraße 33, 04103 Leipzig, Germany; 2 Research Group Primate Behavioural Ecology, Department of Human Behavior, Ecology and Culture, Max-Planck-Institute for Evolutionary Anthropology, Deutscher Platz 6, 04103 Leipzig, Germany; 3 German Center for Integrative Biodiversity Research (iDiv), Leipzig, Germany; 4 Social Evolution in Primates Group, German Primate Center (Deutsches Primatenzentrum), Kellnerweg 4, 37077 Göttingen, Germany; 5 Institute of Biology, Free University of Berlin, Königin-Luise-Str. 1-3, 14195 Berlin, Germany; 6 Research Group of Mass Spectrometry, Institute of Analytical Chemistry, Leipzig University, Linnéstraße 3, 04103 Leipzig, Germany

**Keywords:** Olfactory communication, Anogenital swelling, Menstrual cycle, Olfactory fertility cue, Gas chromatography–mass spectrometry, Ecology, Ecology, Neuroscience, Physiology, Zoology

## Abstract

To attract mating partners, female mammals communicate their reproductive status through one or multiple sensory modalities, providing redundant or complementary information. Chimpanzees (*Pan troglodytes*) are an excellent model for studying multimodal communication. Exaggerated sexual swellings of females serve as visual proxy for ovulation but their temporal imprecision suggests that olfactory cues may exist in addition to swellings to pinpoint fertility more accurately. Here, we combined gas chromatography–mass spectrometry, hormonal analyses, and two-choice sniffing bioassays to examine (1) whether female anogenital odours change during the fertile period, and (2) whether males are able to detect these changes by odours alone. Our chemical results suggest that complementary changes in chemical profiles linked to menstrual cycle phases exist in addition to prominent changes of anogenital odours associated with swelling stages, even if they are statistically less strongly supported. Male responsiveness in behavioural bioassays was too low to draw conclusions regarding their ability to detect these subtle shifts when presented with anogenital odours only. Overall, our chemical analyses of female anogenital odours suggest the existence of a multimodal fertility cue in chimpanzees, wherein visual signals seem to be complemented by subtle changes in odours’ chemical profiles associated with menstrual cycle phases.

**Supplementary Information:**

The online version contains supplementary material available at https://doi.org/10.1038/s41598-026-70687-z.

## Introduction

Mating success is a fundamental part of individual fitness. To attract mating partners, female mammals communicate their reproductive status through various sensory modalities, including vision, acoustics, and/or olfaction^1,2^. Sexual selection exerts strong pressures on traits that improve inter-sexual communication^2^. Such communication may involve a single modality, but highly social, group-living animals often engage in multimodal communication^3,4^. For example, many species of birds show complex visual displays during singing^5–7^, and some bats combine vocalizations with fanning odours toward potential mates^8^.

Primates are remarkable in the diversity of sensory modalities used to communicate their reproductive status^9^. Some indicators of reproductive status may constitute *signals*, i.e., traits that evolved to communicate fertility-related information to conspecifics, or *cues*, i.e., traits that did not evolve for communicative purposes, e.g., being by-products of hormonal changes^10^. Female primates can display visual cues, such as sexual swellings in chimpanzees (*Pan troglodytes*)^11^ and olive baboons (*Papio anubis*)^12,13^ or sexual skin colour in rhesus macaques (*Macaca mulatta*)^14^. Some primates use auditory cues, such as copulation calls in Barbary macaques (*Macaca sylvanus*)^15^, or behavioural cues, such as solicitations and other proceptive behaviours in black howler monkeys (*Alouatta pigra*)^16^ and long-tailed macaques (*Macaca fascicularis*)^17^. Finally, female primates use olfactory cues, such as scent marks in common marmosets (*Callithrix jacchus*)^18^ and ring-tailed lemurs (*Lemur catta*)^19^. These cues can be exhibited in various combinations, whereby different components may transmit redundant or complementary information^20^.

Whereas visual and acoustic cues of fertility have been well-studied in non-human primates, recent studies also highlight the importance of smell, a modality which had long been neglected compared to other senses in primate communication studies^21–23^. Chemical analyses of female body odours demonstrated differences between cycling and non-cycling females (ring-tailed lemurs)^19^, different phases of the menstrual cycle (common marmosets^18^; Japanese macaques, *Macaca fuscata*^24^; olive baboons^25^), and stages of sexual swelling (chimpanzees)^26^. Some studies identified specific chemical compounds that are related to fertility^18,24–26^. Behavioural studies further demonstrated that female body odours can convey fertility-related information to males. In several observation studies, olfactory inspection of the female anogenital region by males increased during the fertile period (olive baboons and chacma baboons, *Papio ursinus*^27,28^). In behavioural tests, males of several primate species reacted to body odours collected from fertile females with increased exploratory and arousal behaviours (common marmosets^18,29^; cotton-top tamarins, *Saguinus oedipus*^30^), as well as with an increase in testosterone levels (common marmosets^31^; stumptailed macaques, *Macaca arctoides*^32^).

Chimpanzees (*Pan troglodytes*) represent an excellent model for investigating whether olfaction integrates with other sensory modalities to communicate fertility multimodally. Female chimpanzees exhibit several cues that may indicate fertility with different levels of precision. Females have exaggerated sexual (anogenital) swellings, which vary in size across the menstrual cycle phases but are not precisely aligned with them^33^. The period of maximal swelling lasts for about one third of the menstrual cycle (ten to twelve days on average, with the length of the menstrual cycle averaging 36 days) and includes parts of the follicular and periovulatory phases^33–35^. Ovulation occurs within the period of maximal swelling, and although the size of the swelling slightly increases as ovulation approaches, the precise ovulation day cannot be determined from the swelling size alone^36,37^. Females also exhibit proceptive and resistance behaviours at different rates during fertile and non-fertile periods, with higher proceptivity and lower resistance to preferred males during the fertile period compared to non-fertile period^38^. Finally, females produce copulation calls, although their rates or acoustic structure do not vary between fertile and non-fertile periods^39,40^. None of these cues, however, provides precise information about the fertile period, and it has been suggested that concealing ovulation allows females to prevent monopolization by dominant males and thereby confuse paternity and reduce infanticide risk^40,41^. In line with this, although copulations occur most frequently during maximal swelling, mating also occurs outside this period^42^.

Despite this presumed imprecision of fertility cues, observations suggest that male chimpanzees might get more precise information about fertile days from olfactory cues than from visual cues (swelling size) alone. First, males sniff at females more often during maximal swelling^43^. Second, males display increased mating interest during the periovulatory period compared to other days of maximal swelling^36,44^. As male competition over the females is intense, with high-ranking males gaining most of the copulations^45–47^, males should benefit from using precise fertility indicators, as it would allow them to maximize mating efforts on fertile females. A potential mechanism for olfactory fertility cues may involve cycle-dependent hormonal changes. Since levels of steroid hormones vary in relation to sexual swelling stage as well as menstrual cycle phase^48,49^, hormone-related olfactory changes may correspond to both processes.

Previous studies in chimpanzees have found that some volatile fatty acids in female vaginal secretions change in levels across the menstrual cycle^50,51^. A more recent study, showed that chemical composition of body odours differs between swelling stages and found especially compounds that were either most or least intense during the maximal swelling stage^26^. However, this study investigated body odours (i.e., from torso and limbs) instead of anogenital odours that are more often investigated by males^43^ and had no hormone samples available to relate changes also to menstrual cycle phases. Thus, it remains unclear whether chemical profiles of anogenital odours vary between menstrual cycle phases, in addition to variation across swelling stages, and whether this variation provides more precise information about fertility than the swelling size alone. Here, we used information not only on swelling stages, but also on the menstrual cycle phases, obtained from hormonal analyses, to investigate both of their effects on the chemical composition of female anogenital odours. In addition, following an integrative approach, we have also carried out behavioural tests to assess whether the scent alone is already sufficient to evoke increased interest when it originates from a periovulatory phase. Thus, we investigated male perception of female anogenital odours by presenting males the odours from unfamiliar females in various menstrual cycle phases. Our study had two aims: (1) to unravel the impact of sexual swelling stage and menstrual cycle phase on the chemical composition of anogenital odours of female chimpanzees; and (2) to examine whether male chimpanzees can distinguish between the anogenital odours from unfamiliar females in different phases of the menstrual cycle. We predicted that the chemical composition of female odours will vary across the menstrual cycle phases, especially during the periovulatory phase compared to non-fertile phases, in addition to the variation between the swelling stages, and therefore provide more precise information on fertility than the swelling alone. Moreover, we expected that males will display more investigations towards odour samples from females during their periovulatory phase compared to non-fertile phases, reflecting their ability to detect cycle-related differences by anogenital odours alone.

## Methods

### Sampling

**Fig. 1:**
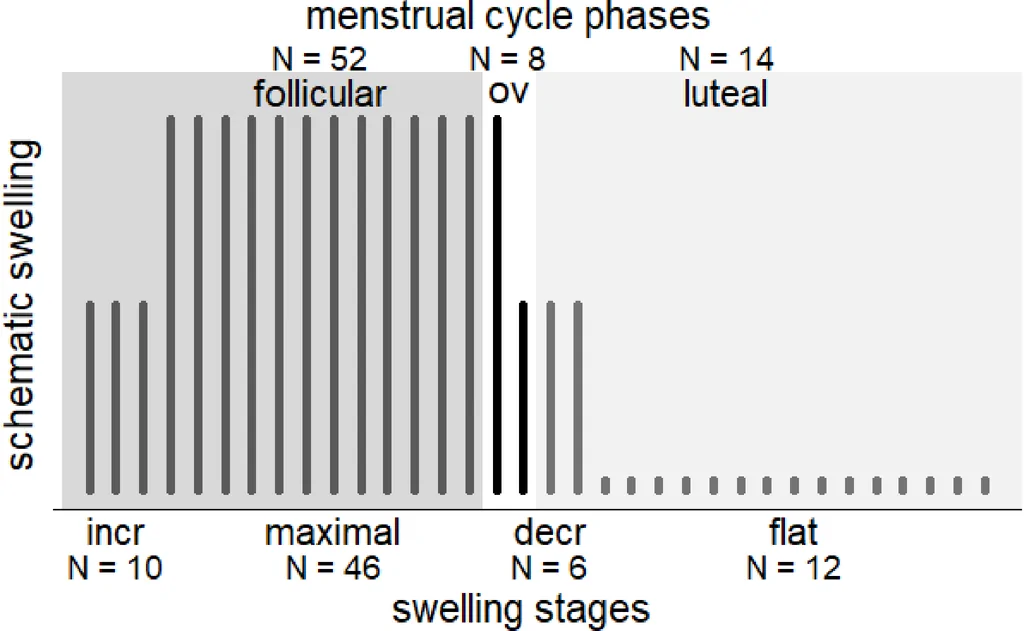
Schematic representation of swelling stages using dark bars (small = flat, medium = increasing/decreasing, large = maximal swelling) reflecting the average cycle length over all females (increasing: 3 days, maximal: 13 days, decreasing: 3 days, flat: 15 days). Sample distribution across the menstrual cycle phases is shown above the graph and across the swelling stages below. The periovulatory window (”OV”) is shown as white area.

Samples were collected by L.S. in June and July 2022 at Zoo Dudley (Dudley, UK) from five naturally cycling female chimpanzees aged 27 to 47 years (mean ± SD: 38.8 ± 9.1) living in a social group of seven females. The animals were trained using positive reinforcement to voluntarily participate in sampling. Samples were collected over one complete sexual swelling cycle (mean ± SD: 34 ± 2.5 days), from the onset of increasing swelling to the next. We differentiated four swelling stages: flat, increasing, maximum, and decreasing, following^34^ (Fig. 1).

We collected *saliva samples for hormone analysis* using synthetic swabs (Salivette^®^ Cortisol, Sarstedt, Germany). Females were trained to take swabs directly with their mouth, chew for at least ten seconds, and return the chewed swab by presenting it between the teeth. The saliva was retrieved by manually centrifuging the swabs. Swabs were stored at − 18 °C during sample collection and at − 80 °C for storage. In parallel, we collected *odour samples for chemical analysis and bioassays* from the anogenital region since a previous study found that males mainly sniffed at this area compared to other body parts or body fluids^43^. Animals were trained to present their anogenital region to the grid of the enclosure for sampling. We used different methods for gas chromatography–mass spectrometry (hereafter, GC–MS) analysis (optimized for volatile preservation) and bioassays (optimized for volatile release during bioassays), both successfully used in this combination in previous studies^18,52^.

*Odour samples for chemical analysis* were collected using preconditioned thermal desorption (TD) tubes (stainless steel, 1/4 in. × 3 1/2 in., Supelco, Bellefont, USA) containing Tenax TA (0.095 g) and XAD-2 (0.21 g, Sigma-Aldrich, Steinheim, Germany, see^18^ for details) from close to the skin (about 1–2 cm away). A BiVOC2 pump (Umweltanalytik Holbach GmbH, Germany) was used to draw 0.5 L air through pre-cleaned TD tubes with a flow of 1 L/min for 30 s. Afterwards, TD tubes were closed with Swagelok Brass caps (1/4 in., Supelco, Bellefont, USA) and stored at room temperature. In addition to the actual samples, we included three types of blank samples: environmental blank samples, collected in front of the enclosures in the absence of animals (*N* = 10), TD tubes present at Zoo Dudley during the sampling period but were never opened (*N* = 5), and several laboratory blanks with empty Tenax-filled TD tubes that remained in the lab and were analysed between sample measurements.

To collect *odour samples for bioassays*, we dabbed sterile gauze pads (viscose and polyester, Premier Healthcare & Hygiene Ltd, Great Britain) on the anogenital area. In more detail, we pressed one piece of gauze pad repeatedly onto a single area of approximately 4 cm² of the swelling of the individual until a total contact time of 20 s. Most dabs were placed in the middle of the swelling (73% of all samples). Depending on the positioning of the individuals, some samples also covered parts from the bottom (34%) or the front-facing part of the swelling (16%). We stored samples in 4 mL glass vials at − 18 °C during sample collection and at − 80 °C for storage. We used bioassay samples from the days included in the chemical dataset. Due to an unexpected 2.5-year delay in exporting samples from Zoo Dudley to Leipzig University, we collected additional samples for bioassays for comparison. They were collected over one complete cycle according to the original dataset from two non-contracepted and naturally cycling females aged 28 and 34 years living in one non-breeding group of five females and one sterilised male at the Wolfgang Köhler Primate Research Centre (WKPRC) in the Zoo Leipzig, Germany, in October and November 2025 following the same protocol.

### Ethics

The study was ethically approved by the European Association of Zoos and Aquaria (EAZA), especially the EAZA Ex situ Programmes (EEPs) for Chimpanzees as well as sample collection by the Zoo Dudley, UK. We obtained and transported samples under CITES export and import regulations in compliance with relevant national and international regulations (export permit no: 24GBEXPR4E6RF; import permit no: DE-E-05276/24) and transferred them under import permit issued by the Saxon State Ministry for Social Affairs and Social Cohesion.

Sample collection at the Wolfgang Köhler Primate Research Centre (WKPRC) in the Zoo Leipzig, Germany was in accordance with the legal requirements of Germany and approved by the Ethics committee of the Zoo Leipzig and the Department of Comparative Cultural Psychology, Max Planck Institute for Evolutionary Anthropology in Leipzig, Germany.

### Hormone analysis

Salivary progesterone concentrations were measured in the endocrinology laboratory of the German Primate Centre (Göttingen, Germany) in March 2025 for samples from Zoo Dudley and in December 2025 for samples from Zoo Leipzig using an enzyme immunoassay (EIA) based on the monoclonal antibody CL425 (Quidel clone no. 425, final purification by C. Munro, Davis, CA). Details of the analysis are described in Supplementary Material. Progesterone levels measured from saliva presumably reflect the hormonal state at the moment of sampling with only a slight time delay (20–30 min after appearance in blood)^53^ and were used, in conjunction with an assessment of the swelling, to classify the phases of the menstrual cycle, whereas menstrual bleeding could not be reliably observed and taken into account.

This enabled us to classify samples taken during the two days immediately before the rise in progesterone level (two standard deviations above average of the five days before) as belonging to the periovulatory phase. We classified samples taken during the flat phase (after the progesterone rise with time gap until next tumescence) to be luteal and samples collected during the increasing and maximal swelling stages (before the periovulatory window) to be follicular (Fig. 1). Samples that fell between swelling stages or cycle phases (i.e., first day of maximal swelling, three days before and one day after periovulatory window) were excluded, resulting in a total of 74 samples (see Fig. 1 for sample distribution across cycle phases and swelling stages; 12–17 samples per female, mean ± SD: 14.8 ± 2.2).

### GC–MS analysis and chemical profiling

We shipped TD tube samples to Leipzig University immediately after collection and measured in September 2022 using a GCMS-TQ8040 (Gas Chromatograph GC-2010 Plus and a Triple Quadrupole Mass Spectrometer, Shimadzu, Kyoto, Japan; see details in Supplementary Material). Chemical data were analysed using an established, semi-automated procedure^18^ using AMDIS 2.73^54^ (see details in Supplementary Material). The final chemical data set included 200 compounds for which we calculated relative intensities (peak area / sum of all peak areas x 100) to account for differences in sample intensities.

### Bioassays

We conducted bioassays at Tacugama Chimpanzee Sanctuary (Freetown, Sierra Leone) from January to February 2026 (J.W.S.). The veterinary department of the Tacugama Chimpanzee Sanctuary, Sierra Leone, reviewed and ethically approved our protocols for bioassays. Odour samples of unfamiliar females were presented to 18 male chimpanzees kept in different groups. Only 13 males (aged 8 to 42 years; mean ± SD: 18.5 ± 8.8) voluntarily participated in at least one test.

Thawed swab samples were placed in the setup (pre-cleaned with 70% ethanol, wearing gloves) attached to the enclosure’s grid and allowing to present the samples in two open glass vials set 56 cm apart and facing the subjects (see details on storage, thawing and the setup in Supplementary Material). Samples remained in the setup for five minutes in presence of males. To minimize experimental bias, the experimenter was visually obscured, trials were unrewarded to prevent food conditioning, and consecutive tests with the same male were separated by at least four days (see details of bioassay implementation in Supplementary Material). Male behaviour was video-recorded for subsequent analysis.

We conducted three types of tests in two-choice tasks in randomised order: samples from females in (1) follicular vs. periovulatory phase (N_males_ = 16), (2) luteal vs. periovulatory phase (N_males_ = 18) and (3) blank swab (sterile swab) vs. female sample (N_males_ = 19) (see more details in Supplementary Material). Bioassays were designed and conducted as a full-scale study, with the aim of presenting all males with all three test types to obtain balanced and robust results. However, males participated only in part of our trials, resulting in a lower sample size than anticipated. As two males successfully participated twice under the same condition (luteal vs. periovulatory), we averaged their measurements to ensure independent observations for analysis. This resulted in the following sample sizes: four males for follicular vs. periovulatory tests, six males for luteal vs. periovulatory tests, and nine in blank tests. Due to low sample size, results are discussed descriptively.

Video analysis was conducted blindly to sample identity. We evaluated the number of investigation approaches and the total investigation duration. Investigating included (1) sniffing (bringing the nose within four centimetres of the sample or touching the mesh in front of the sample and sniffing at the finger within three seconds); (2) licking (dipping the tongue or open mouth to the mesh in front of the sample). To control for an observer bias, a second observer coded twelve videos. An intra-class correlation (ICC) was calculated using the R package *irr* v. 0.84.1^55^, showing a high to excellent consistency between observers (ICC = 0.963 for number of investigations and ICC = 0.855 for investigation durations).

### Statistical analysis

#### General statistical approach

**Fig. 2:**
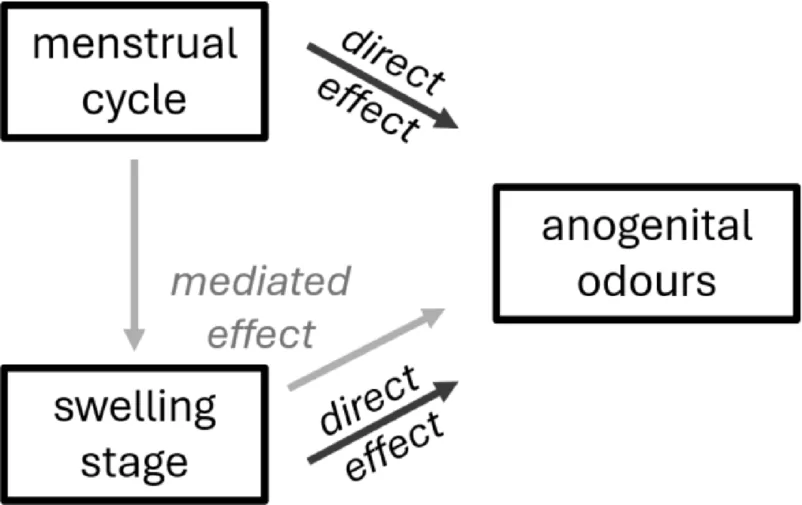
Proposed simplified directed acyclic graph illustrating the hypothesized direct effects of menstrual cycle phase and swelling stage on anogenital odours and the hypothesized effect of menstrual cycle phase mediated by swelling stage.

As swelling stage might mediate the effect of menstrual cycle phase (Fig. 2), we aimed to separate their direct and total (direct + mediated) effects. Since swelling stage is physiologically linked to menstrual cycle phase and expected to mediate rather than modulate menstrual cycle effects, we did not model an interaction between the two predictors. Instead, we conducted two analyses: (A) including both predictors in the same model to assess the direct effects for both; and (B) including only menstrual cycle phase to assess its total effect (see more details in Supplementary Material). We conducted all statistical analyses using R v. 4.4.2 (R Core Team, 2025).

### Chemical composition

We fitted generalized linear mixed models (GLMMs) using the package *lme4* v. 2.0–1^56^ to investigate whether sexual swelling stage and menstrual cycle phase influence chemical composition of anogenital odours. For both variants (A) and (B), the vectorized data matrix was composed of all compounds (*N* = 200) for all odour samples (*N* = 74), resulting in 14,800 compound intensities as response. Each model variant was fitted with two response transformations: (1) normalised and (2) log-transformed intensities. Normalisation centres all compound intensities to a mean of zero and a standard deviation of one, equalising the contribution of all compounds and *increasing sensitivity on difference of compounds among samples.* Log-transformation (log(x + 0.001) and arcsine) reduces the over-influence of the largest compounds^57^, but maintains the intensity differences of compounds within samples and is *more sensitive to changes in condition-specific compounds*.

We fitted sexual swelling stage (four factor levels: ‘flat’, ‘increasing’, ‘maximal’, ‘decreasing’) and/or menstrual cycle phase (three factor levels: ‘follicular’, ‘periovulatory’, ‘luteal’), the enclosure type (three factor levels), and GC–MS batch (three factor levels) as fixed effects. We included rows and columns (samples and compounds) of the vectorized data matrix as random effects to account for pseudoreplication and heteroscedastic variance^58^. Moreover, we added identity (ID, five factor levels) of the females of Zoo Dudley as additional random effect. We included all meaningful random interactions (swelling stages and cycle phases within ID) and fitted random interactions of sexual swelling stages and/or menstrual cycle phases within compounds as test predictors and the random interaction of GC–MS batch within compounds to get more reliable p-values. We estimated a random intercept for each compound × swelling stage/menstrual cycle phase combination, allowing to assess *differences between stages/phases for each compound*. Using likelihood ratio tests (LRT)^59^, we compared full models against null models lacking random interactions (see model overview in Supplementary Material). If full-null model tests were significant, we compared full models against reduced models excluding random interactions of each predictor separately, a method that has performed well for detecting effects of predictors on chemical compounds in a simulation study (Weiß et al. in press).

We found no violations against the assumptions of normal distribution, homogeneity of residuals and residuals against fitted values for all models. Variance inflation factors, determined using the R package *car* v. 3.1-3^60^, did not exceed 2.0, indicating no collinearity issues. We assessed model stability by sequentially excluding individual levels of the random effects, refitting the full model to each resulting subset, and comparing the parameter estimates with those of the full model, indicating good model stability. To confirm that our results are robust, although they are based on a dataset containing considerably more follicular samples than periovulatory and luteal samples, we also analysed a reduced dataset with a more limited selection of follicular samples (*N* = 25 instead of 52) with the same statistical analyses, which fully confirmed our findings (see Supplementary Material).

### Chemical profiles

We performed permutational analyses of variances (perMANOVA) in R package vegan v. 2.6-8^61^ to investigate the effects of menstrual cycle phase and swelling stage on whole chemical profiles of odours. As above, we used two variants (A) and (B) and two response transformations, normalised and log-transformed. The perMANOVA was performed on Bray–Curtis dissimilarities, with individual ID included as a strata term.

### Compound identification

When a GLMM revealed significance, we investigated the differences for each compound between the levels of the test predictors. Following^62^, we considered compounds with differences of 2.5 standard deviations above the average of all 200 compounds to be of particular importance. We tentatively identified these compounds via library comparison using NIST14 Mass Spectral Library (National Institute of Standards and Technologies, Gaithersburg, MD, USA), accepting hits with a similarity index above 80 and excluding known contaminations. We found three of the most affected substances in our internal reference library and thus directly confirmed the library hit by comparison to standards. In addition, we confirmed all suggested identifications by comparing retention indices from NIST to those of a known alkane series measured on the same instrument using the same method (see Supplementary Material for further details on identification).

## Results

### Chemical analyses

#### Chemical composition

**Fig. 3:**
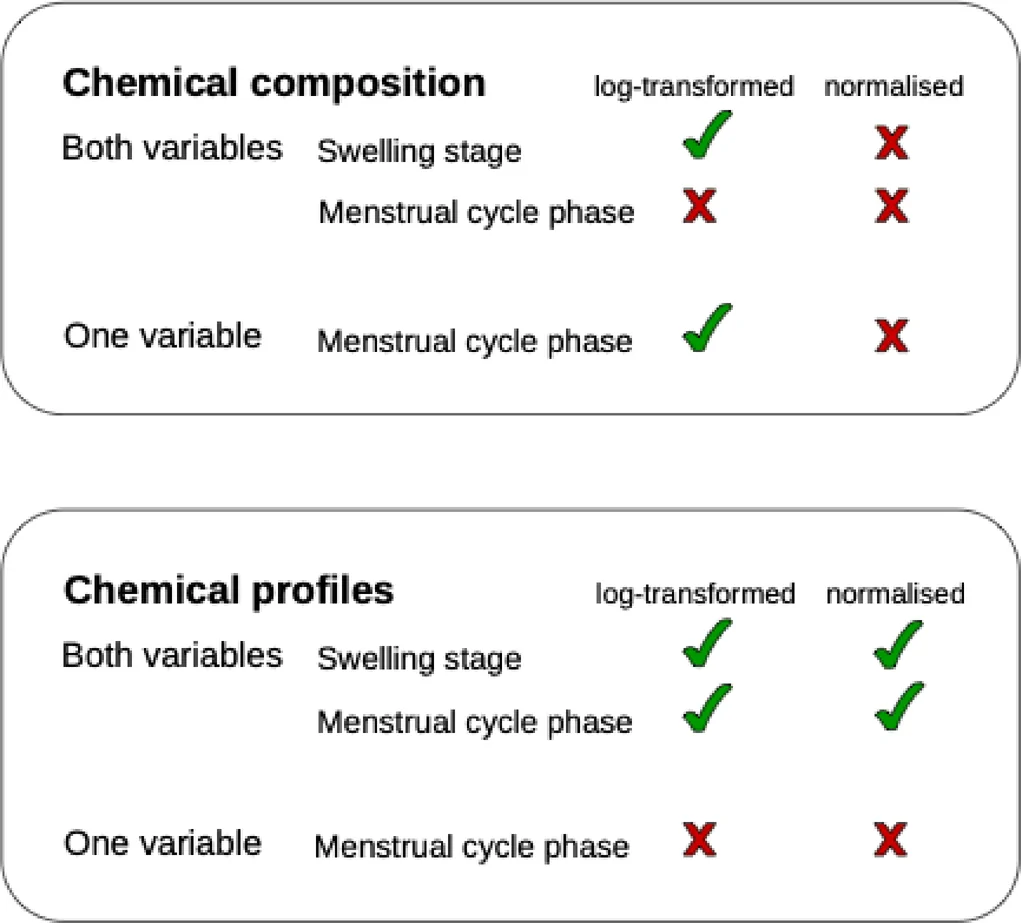
Summary of results. Green checks indicate a statistically significant effect; red crosses indicate the absence of significance.

When *both* variables were included (LRT full-null: χ2 = 65.9, df = 2, *p* < 0.001), we found a direct effect of swelling stage (LRT swelling: χ2 = 54.3, df = 1, *p* < 0.001), but not of menstrual cycle phase (LRT cycle: χ2 = 0, df = 1, *p* = 0.999) on chemical compounds in the log-transformed model variant (Fig. 3). When the response was normalised, neither swelling stage nor menstrual cycle phase had a significant effect (LRT full-null: χ2 = 0, df = 2, *p* = 1).

**Fig. 4:**
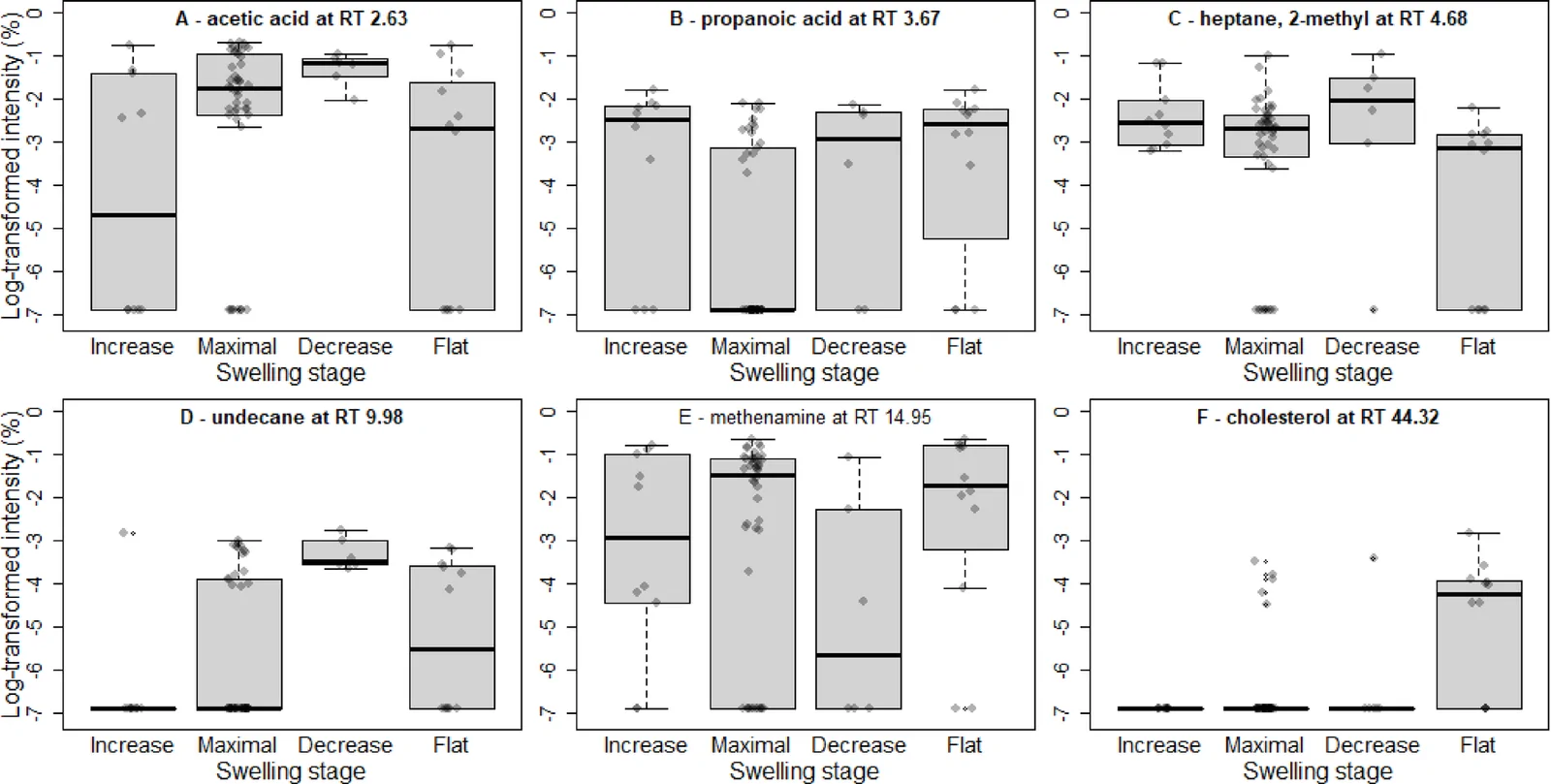
Compounds most affected by swelling stage or menstrual cycle phase shown across swelling stages (log-transformed response). Only the compounds with names in bold were found to be most affected by swelling stage (largest differences between swelling stages > 0.58, i.e., the average differences + 2.5 SD of all compounds for swelling stage). Differences between most distinct swelling stages were for A: 0.66, B: 0.90, C: 0.63, D: 0.66 and E: 0.66. Boxplots show medians and first and third quartiles, grey dots represent raw data points.

**Fig. 5:**
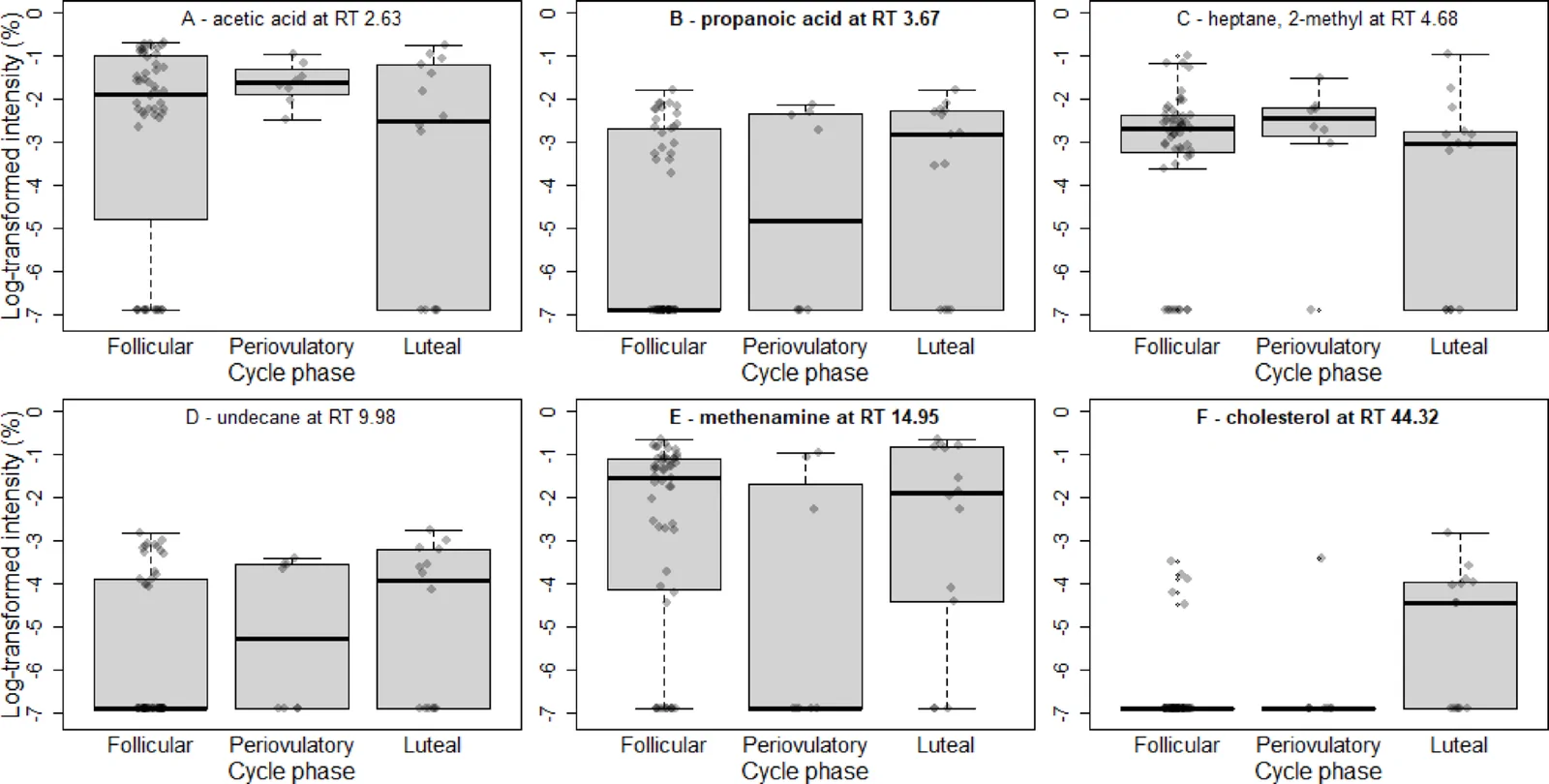
Compounds most affected by swelling stage or menstrual cycle phase shown across menstrual cycle phase (log-transformed response). Only the compounds with names in bold were found to be most affected by menstrual cycle phase (largest difference between menstrual cycle phases > 0.27, i.e., the average differences + 2.5 SD of all compounds for menstrual cycle phase). Differences between most distinct menstrual cycle phases were for B: 0.32, E: 0.36 and F: 0.35. Boxplots show medians and first and third quartiles, grey dots represent raw data points.

When we included *only menstrual cycle phase*, it significantly affected chemical composition (LRT: χ2 = 11.6, df = 1, *p* < 0.001) with log-transformed, but not with normalised response (LRT: χ2 = 0, df = 1, *p* = 1). Based on the individual differences per compound with log-transformed response, we found five compounds to be particularly affected by swelling stage and three compounds by menstrual cycle phase (differences 2.5 SD > average; see Supplementary Table S2 for substance overview and Figs. 4 and 5). Propanoic acid and cholesterol were affected by both, swelling stage and menstrual cycle phase. Propanoic acid was least intense during the maximal swelling phase compared to the other three swelling stages and cholesterol was most intense during the flat phase compared to the other three swelling stages. Accordingly, both were most intense during the luteal phase compared to the other two cycle phases. Acetic acid, 2-methyl heptane and undecane were affected only by swelling stage. Acetic acid and 2-methyl heptane were most intense during maximal swelling and/or decreasing swelling compared to the swelling stages before and afterwards. Undecane had high intensities during increasing, maximal and decreasing swelling compared to the flat phase. Methenamine was affected only by menstrual cycle phase and was less intense during the periovulatory cycle phase compared to luteal and follicular phase.

#### Chemical profiles

When *both* variables were included, swelling stage as well as menstrual cycle phase significantly affected whole chemical profiles with log-transformed (swelling: R² = 0.09, F = 2.59, *p* = 0.004 and cycle: R² = 0.05, F = 1.91, *p* = 0.046) and normalised response (swelling: R² = 0.09, F = 2.20, *p* = 0.004 and cycle: R² = 0.06, F = 2.14, *p* = 0.010). When *only menstrual cycle phase* was included, it affected the profiles neither with the log-transformed (R² = 0.03, F = 1.21, *p* = 0.248) nor with the normalised response (R² = 0.04, F = 1.34, *p* = 0.104).

### Bioassays

**Fig. 6:**
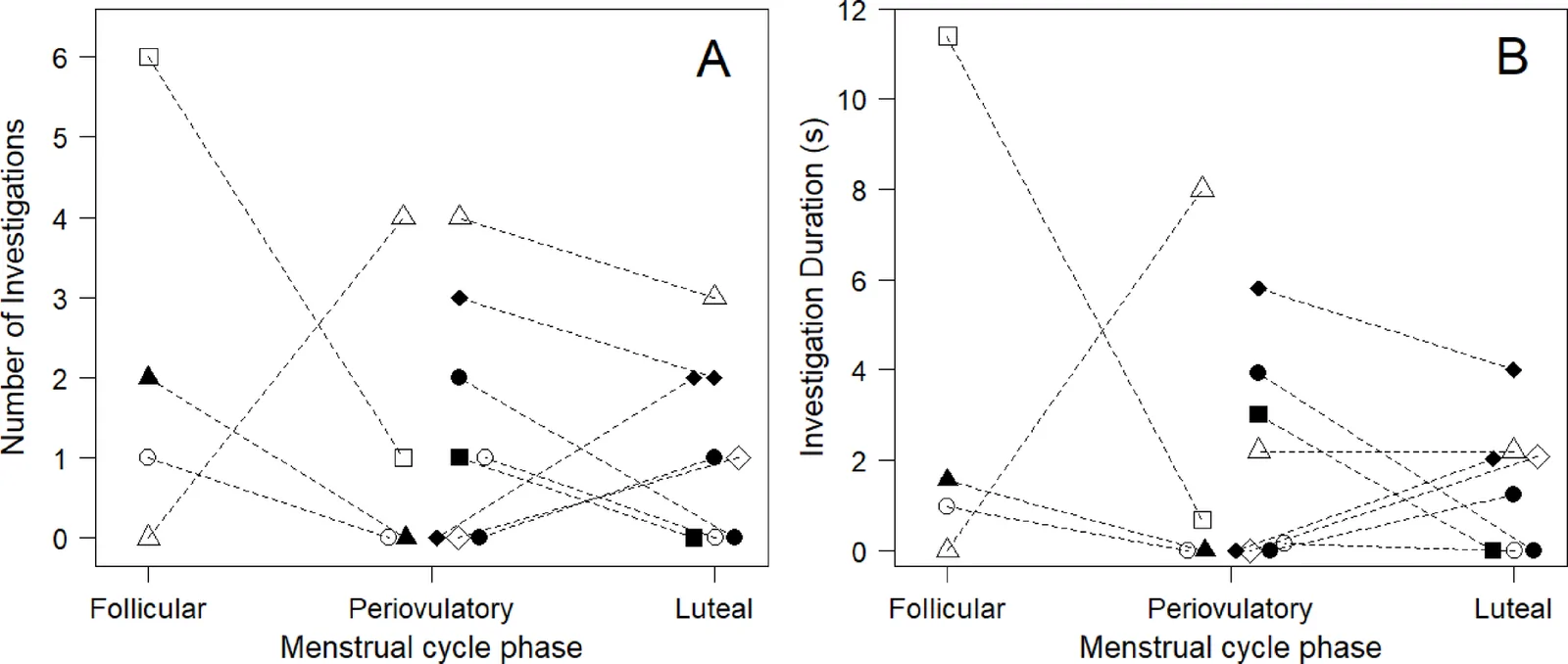
Investigation numbers (**A**) and durations (**B**) of odour samples from different menstrual cycle phases by males. Each symbol represents one male individual (two males are represented twice for luteal vs. periovulatory, with a black circle and a black diamond), dotted lines connect data points of the same behavioural test run.

Within our behavioural tests, males showed interest in our setup in only 21 of 53 cases. In most blank tests males investigated female samples more often than blanks (seven of nine tests, N investigations: mean blank ± SD: 3.11 ± 4.04 vs. mean sample ± SD: 5.00 ± 4.66) and investigation duration was longer for samples than for blanks (six of nine tests, investigation durations: mean blank ± SD: 4.21 ± 4.47 vs. mean sample ± SD: 8.56 ± 8.28, N_males_ = 9). When presenting samples from follicular vs. periovulatory phase, only one of four males investigated the periovulatory sample more often and longer (N investigations: mean periovulatory ± SD: 1.3 ± 1.9 vs. mean follicular ± SD: 2.3 ± 2.6; investigation durations: mean periovulatory ± SD: 2.2 ± 3.9 s vs. mean follicular ± SD: 3.5 ± 5.3 s, N_males_ = 4, see Fig. 6). For the comparison of samples from luteal vs. periovulatory phase, four of six males investigated periovulatory samples more often (N investigations: mean periovulatory ± SD: 1.4 ± 1.4 vs. mean luteal ± SD: 1.1 ± 1.2) and three of six males investigated periovulatory samples longer (investigation durations: mean periovulatory ± SD: 1.7 ± 1.3 s vs. mean luteal ± SD: 1.3 ± 1.3 s, N_males_ = 6, see Fig. 6).

## Discussion

Chemical composition of female anogenital odours varied across both sexual swelling stages and menstrual cycle phases. While the swelling stage had a significant direct effect on odours in most statistical analyses, an additional direct effect of the menstrual cycle phase extending beyond the effect of the swelling stage was subtle and could be revealed only in some analysis variants. Contrary to our expectations, we did not observe a male preference towards odours of fertile females. However, male interest toward the odour stimuli was generally too low to draw reliable conclusions about their ability to discriminate between odours from different phases of the menstrual cycle.

We hypothesized that olfactory cues provide more precise information about the timing of ovulation than the swelling size alone building on evidence that swelling size serves as a proxy for ovulation, combined with observations that male chimpanzees sniff at females during their maximal swelling and display increased mating interest during the periovulatory period^36,43,44^. Our chemical results provide partial support for this hypothesis. First, we examined whether individual compounds change across swelling stages and/or menstrual cycle phases. Swelling stage had a significant direct effect on anogenital odours, corroborating our previous findings of odours from other body parts being affected by swelling stages^26^. The menstrual cycle phase had a significant effect only when mediated by swelling stage but did not reveal a direct effect. The effects were significant only for log-transformed response, focused on differences between samples, but not for normalised response, focused on compound differences within samples.

Second, we examined whether whole chemical profiles change across swelling stages and/or menstrual cycle phases. Here, swelling stage had a direct effect on odour profiles, revealed in both log-transformed and normalised responses. Additionally, the menstrual cycle phase had a direct effect on odour profiles, extending beyond swelling and consistent across both response transformations. The overall chemical profiles, therefore, are influenced by both factors independently. These findings suggest that an olfactory signature of the menstrual cycle provides some *complementary* information about the timing of the periovulatory period. However, both effects were relatively small (swelling stages: 9% variance explained, menstrual cycle phases: 5–6%) and statistical support was stronger for swelling-related changes. Interestingly, the small effect sizes are roughly in line with the cycle phase differences of 6% found in vaginal odour in a study on Japanese macaques^63^, although direct comparisons should be made with caution.

The influence of swelling stage and menstrual cycle phase on odour was more consistent for the log-transformed response across all analyses. As the log-transformation allows compound intensities to differ in relation to the remaining chemical profile of a given sample, these findings suggest that fertility-related information could be encoded within odour chemical composition at a single time point. Consequently, males may be able to assess a female’s reproductive status through a single investigation, without a need for continuous odour monitoring. Our evidence for olfactory changes with log-transformed response would be consistent with the dynamics of a fission-fusion society, where subgroup composition may change daily and males may lack consistent access to specific females. Furthermore, this would allow lower-ranking males, who may be socially precluded from consistent access to fertile females, to still obtain fertility cues during opportunistic encounters. However, in the analysis of the whole chemical profiles, the effects of swelling stage and menstrual cycle phase on odour were also found for the normalised response. Since with normalised response, affected compounds might rather reflect changes over time, these findings indicate that additional fertility cues may be available to males continuously monitoring the same female, either over the course of her menstrual cycle or, based on visual signals, over her maximal swelling stage.

In total, six chemical compounds were affected by swelling stage and/or menstrual cycle phase (Figs. 4 and 5). Five compounds were affected by swelling stage, and two of them were additionally affected by menstrual cycle phase, consistent with our finding that menstrual cycle phase does not have a direct effect on odours but is mediated by swelling stage. Compared with the previous study, which examined body odour at different parts (rather than anogenital) in relation to the phases of swelling^26^, we found a different set of compounds. This is not surprising given the different areas of body parts examined. Only cholesterol-related compounds were found in both studies.

Two of the affected compounds, acetic acid and propanoic acid, are aliphatic acids that were previously found in vaginal secretions of chimpanzees^50,51^, humans^64^, and rhesus macaques^65,66^, as well as in scent glands of common marmosets^67^ (see Supplementary Material Table S2). Acetic acid was more abundant during the maximal and decreasing swelling compared to flat and increasing swelling, whereas propanoic acid was least abundant during the maximal swelling compared to the other stages. Moreover, propanoic acid was least abundant during the follicular phase with increasing abundance towards the periovulatory and luteal phase of the menstrual cycle, matching the intensity changes with regard to swelling stage. In previous studies, acetic and propanoic acid, in a mixture together with other short-chain aliphatic acids, were reported to stimulate sexual behaviours in male rhesus macaques and were therefore referred to as sexual pheromones, or “copulins”^65,66^, although these studies were later criticised^68^. The amount of aliphatic acids in vaginal secretions, with acetic and propanoic acids being the most prominent in all species, showed some variation across the cycle in humans and macaques, although the patterns were not consistent^64,66^. In one chimpanzee study, a female with the highest levels of acetic and propanoic acids was the only female followed by a male^51^.

Cholesterol was more abundant during the flat swelling stage compared to the other three swelling stages and in the luteal phase compared to follicular and periovulatory phase. Cholesterol is the precursor for steroid hormones such as oestradiol and progesterone^69^, both of which are known to mediate swelling size. Specifically, increasing swelling is correlated with the rise of oestrogen levels in the follicular phase, whereas decreasing swelling follows a rise in progesterone, which inhibits the effects of oestrogen^33,48,49^. As progesterone levels increase during the flat swelling stage and in the luteal phase^33,69^, the increase in cholesterol levels observed during this period may reflect the need for progesterone synthesis. This is in line with results of the previous study in chimpanzees investigating body odours that also described cyclic variation in cholesterol-derived compounds, which were more abundant during the increasing and decreasing swelling stages compared to the maximal swelling and flat stages^26^, probably reflecting cyclic patterns of processing and utilization of cholesterol for biosynthesis of steroid hormones. Cholesterol itself is not volatile at room temperature and is therefore not expected to be directly detectable via olfaction. However, it is regularly found in analyses of skin odour^70^ and is thus part of the skin substrate which has an effect on the microorganisms growing on it and their metabolic products. Thus, variation in cholesterol levels may nevertheless be associated with changes in other olfactory cues linked to cholesterol metabolism, which may not have been captured by our analytical approach. Importantly, every sampling method introduces its own bias and will, to some extent, capture a different part of the overall odour mixture (e.g.^52^), whilst animal perception will also have a different sensitivity to certain substances. It is therefore important to stress that a single method will not be able to cover the entire spectrum of odour substances. Hence future studies using other sampling methods would be of interest.

Two other affected compounds were alkanes (2-methyl-heptane and undecane) that had previously been found in human breath, saliva, skin, or other tissues^71,72^ and, in the case of undecane, in the sternal scent gland of mandrills^73,74^ (see Supplementary Material Table S2). While they had not been reported in a fertility-related context, their changes across the menstrual cycle may reflect broad physiological changes associated with cyclic hormonal changes^75^. Finally, methenamine, less abundant during the periovulatory phase compared to luteal and follicular phase, has non-mammalian origins and is used as a medication, as well as in production of plastics and cosmetics. Methenamine was previously found in anogenital odours of common marmosets, where its levels were higher in nulliparous females compared to females who gave birth to infants already^18^ and was discussed as potential contamination released through the genital secretions.

Subtle changes in chemical profiles of female odours across menstrual cycle phases, extending beyond swelling stages, could potentially allow females to regulate the access to fertility information to specific males. Visual signals of swelling size are broadcast to all males, while dominant males may be more likely able to approach and smell the female anogenital region. This is corroborated by an earlier finding where the dominant male, when having the choice among several maximally swollen females, preferred the fertile female rather than that with the biggest swelling^36^. These findings align with the graded signal hypothesis that proposes that swellings are probabilistic signals of the timing of ovulation^76^. On the other side, females might also selectively provide information on ovulation probability to specific males in order to balance the benefits of confusing paternity (to reduce the risk of infanticide) with biasing paternity towards preferred males (to increase paternal care and the probability of obtaining ‘good genes’ for their offspring)^76,77^. In line with this hypothesis, an earlier study showed that, despite clear male dominance, female chimpanzees effectively exhibit mate choice by being more proceptive and less resistant to preferred males during the fertile period, resulting in these males having higher mating success irrespective of male rank^38^. From a male perspective, this combination of graded (olfactory) and probabilistic (visual) information could help dominant males to monopolise a female during highest fertility, while still other males will be able to achieve copulations with receptive females during periods of lower ovulation probability.

Similar findings in support of the graded-signal hypothesis and multimodal fertility communication were demonstrated for olive baboons, a species with a social system similar to that of chimpanzees, where females live in multi-male/multi-female groups and mate with multiple males. In this species, too, the visual signals of sexual swellings were shown to be complemented by more precise olfactory information on the timing of fertility window provided by vaginal odours^25^. Similar to chimpanzees, sniffing and copulation rates by dominant male baboons increased during fertile days, suggesting the role of olfactory cues accessible to males at closer range^13,28^. There is also some tentative evidence of urine odour differences between fertile and non-fertile phases in Japanese macaques, another species with a multi-male/multi-female social system, multiple female mating, and visual signals of fertility^24,78^. Olfactory fertility cues, however, are not necessarily accompanied by visual signals, as is evident from research on common marmosets, who lack visual fertility signals but nevertheless exhibit clear fertility-related odour changes^18^.

It remains unclear whether odour changes detected by our chemical analyses are perceivable and relevant to males, because the males participating in bioassays exhibited insufficient interest in the odour samples to allow for a meaningful analysis of preference. We have planned and conducted a full-scale bioassay set-up in which each male had to undergo every trial, in order to test whether they can detect a difference in fertility based on smell alone. However, in addition to low interest in general, successful trials did not reveal any systematic preferences for odours from fertile females. It is possible that olfactory information is only relevant as part of multimodal cues (combined with visual signals and/or behavioural cues). Thus, one conclusion that can therefore be drawn from our results is that future bioassay studies should include both an odour presentation and additional visual swelling stimuli (matching one odour stimulus and mismatching the other).

Particularly in our bioassay runs, however, it is likely that the lack of significant response is also related to methodological limitations that resulted in low motivation and lack of focus in male subjects. Whilst we deliberately chose to carry out the tests at a sanctuary, because there are considerably more male animals present than in zoos and the environmental conditions are ecologically more natural, this may also have introduced some variability into our data collection. First, the volatile composition of the samples may have been compromised by inconsistent storage during fieldwork, including an eight-hour period where temperatures reached 1.5 °C. However, this likely had a negligible impact, as freezing has been shown not to affect odour perception^79^ and the interest in samples did not differ between samples from Zoo Leipzig and samples from Zoo Dudley. During the bioassay tests, males were often distracted by external factors, such as vocalizations and aggressive interactions of animals in the neighbouring enclosures and the calls of wild chimpanzees in the surrounding forest. In some cases, and beyond our control, males were fed directly before testing, which caused inactivity, or found leftover food in the enclosures, which distracted them from the experimental setting. The experimental setup was novel to the animals, and some of them focused on the setup itself instead of odour samples, chewing or tearing at the setup. It is also possible that the lack of previous positive experience of participating in bioassays lowered their motivation to investigate the samples. Consistent with this, the rate of no-sniffing was significantly higher at the Tacugama Chimpanzee Sanctuary, where 32 of 53 trials resulted in a failure to sniff (60%), compared to the Zoo Leipzig pilot study, where chimpanzees are frequently rewarded for test participation and only five out of 30 trials resulted in a failure to sniff (17%). Some of the males were relatively old (up to 45 years), whereby earlier observations suggest that male chimpanzees are still reproductively active at this advanced age^80^. Presented odour samples originated from different animal facilities and females were therefore unfamiliar and not closely related to the males. In this respect, other individual characteristics, such as age, state of health, genetic (dis)similarity, might also be of interest during initial contact, apart from reproductive information. Furthermore, males have no knowledge regarding the females’ baseline odour, which might be necessary in order to perceive differences.

## Conclusions

We predicted that (1) the chemical composition of female odours will vary across menstrual cycle phases in addition to the variation between sexual swelling stages and provide more precise information on fertility than the swelling alone, and that (2) males will be able to detect these differences by odours alone. Our chemical analysis partially supported the first prediction. In addition to the changes in chemical composition as well as chemical profiles with regard to sexual swelling, changes in whole chemical profiles associated with menstrual cycle phases are indicative of some complementary information about the timing of the periovulatory period. However, statistical support was stronger for swelling-related than for cycle-related olfactory changes. The second prediction could not be confirmed as we found no increased interest by males towards odours from the periovulatory period compared to non-fertile periods. Additionally, the low male participation rate in our bioassay tests does not allow us to draw any conclusion about their ability to discriminate between odours from different phases of the menstrual cycle.

Overall, we found that the visual signal of swelling size is mirrored by olfactory changes in anogenital odours. In addition, partial changes in overall chemical profiles also showed direct effects of menstrual cycle phase complementing them and seem to augment previous findings of slight increases in swelling size that indicate approaching ovulation^36^. Thus, our chemical findings support the hypothesis for a multimodal fertility cue in chimpanzees, which should be investigated further, particularly at the behavioural level, using combinations of visual and olfactory cues.

## Supplementary Information

Below is the link to the electronic supplementary material.

Supplementary Material 1

Supplementary Material 2

## Data Availability

The datasets supporting this article have been uploaded as part of the supplementary material.
