# Supplementary material for: Multimodal fertility cues in chimpanzees: how body odours complement sexual swellings
Source: Sci Rep. 2026 Sep 10;16:28363. doi: 10.1038/s41598-026-70687-z (PMC13562712; doi:10.1038/s41598-026-70687-z)
Supplement: Supplementary file 1 [file 41598_2026_70687_MOESM2_ESM.pdf]

METHODS

Hormone Analysis

Progesterone–horseradish peroxidase was used as a conjugate (1). CL425 was raised against 4-pregnen-11-ol-3, 20-dione hemisuccinate:bovine serum albumin and shows broad cross-reactivity with progesterone (100 %) and its metabolites (2). The assay successfully applied to capture cyclical variation serum samples across a range of mammalian species (3), including primates (1), supporting its suitability for assessing reproductive state. Analytical validation in serum has demonstrated high sensitivity (90 % binding = 50 pg mL^{-1}), parallelism between standard curves and serial dilutions of samples, and high precision (intra- and inter-assay coefficients of variation < 10 % and < 15 %, respectively) (4). Parallelism between serially diluted saliva samples and the standard curve was confirmed.

For the assay, saliva samples were diluted with an assay buffer at dilutions of undiluted, 1:2 or 1:3 depending on hormone concentration. Plates were washed prior to use, and 50 µL of either diluted sample or progesterone standard was added to each well, followed by 50 µL of horseradish peroxidase conjugated and 50 µL of antibody solution. After overnight incubation at 4 °C, plates were washed and 100 µL of tetramethylbenzidine (TMB) substrate was added. The reaction proceeded for 45 minutes in the dark at room temperature on a plate shaker. The enzymatic reaction was stopped by adding 50 µL of 2 M sulfuric acid, and optical density was measured at 450 nm with a 630 nm reference filter using a microplate reader. Results are expressed in pg/mL.

All samples and standards were measured in duplicate. Samples with a coefficient of variation exceeding 10 % or falling outside the linear range of the assay were reanalysed. Quality control (QC) samples with known high and low progesterone concentrations were included in each assay run. Inter-assay coefficients of variation (N = 8) were 10.1 % (high QC) and 5.4 % (low QC).

GC–MS Analysis and Chemical Profiling

Using a thermal desorption system TD-20 (Shimadzu, Kyoto, Japan), samples were desorbed at 250 °C for 8 min to a Tenax TA filled cold trap (−20 °C) with a helium flow of 60 mL/min in split mode (split ratio 5). The injection into the gas chromatograph was performed at a pressure of 140.2 kPa by heating the cold trap to 250 °C for 1 min. One-dimensional chromatography was done using two consecutively connected columns (Rxi-1ms: 30 m length, 0.25 mm ID, 0.25 µm film, and SGE Analytical Science BPX50: 2 m length, 0.15 mm ID, 0.15 µm film, Restek GmbH, Bad Homburg vor der Höhe, Germany) with helium 5.0 (Air Liquide, Düsseldorf, Germany) as carrier gas at a flow rate of 1.58 mL/min. The temperature program started at 35 °C for 0.5 min and ended at 320 °C for 25 min after a rise of 6 °C/min. Electron impact ionization was conducted at 70 eV and a source temperature of 220 °C. Mass spectra were acquired over a scan range of m/z 30 – 500.

Chemical data were analysed using an established, semi-automated procedure (5) using AMDIS 2.73 (6) for peak picking to create an aligned list of compounds present in the samples and integrating compounds using GCMSsolution v. 4.20 (Shimadzu, Kyoto, Japan) to get compound intensities. The resulting data included only compounds with a signal-to-noise ratio

(S/N) above 1.0 and were cleaned by removing incorrectly integrated compounds outside the desired retention time range, that occurred in less than 5 % of the samples and that were more intense in blank samples than animal samples. Additionally, known contaminations were excluded after NIST identifications. Retention indices for the library hits taken from the NIST database were compared with retention indices from a series of alkanes (C8, C10, C12, C25, C29, C33) in order to accept the proposed identification where there was a plausible agreement. Three of the library hits were present in the existing internal reference database with standards that had been measured on the same instrument and using the same method. These compounds could therefore be confirmed by direct comparison with reference standards (see Table S2).

Bioassays

We developed and tested the bioassay setup at the WKPRC with male chimpanzees trained to these sets of tests. However, the WKPRC at that time had only 2 adult males available. Therefore, the preference tests using swab samples were conducted at Tacugama Chimpanzee Sanctuary (Freetown, Sierra Leone).

We transported frozen swab samples to Tacugama in a vacuum bag with cooling packs and phase-change elements (delta T GmbH, Fernwald, Germany) and stored them at −6.18 °C (± SD: 2.8 °C). A temperature tracker (ThermoScan 2S, delta T) continuously recorded the temperatures and detected one rise above the freezing point to 1.5 °C for approx. 8 h during a power cut which we considered not critical for the testing success, since repeated thawing-cycles do not seem to have a major impact on sample quality (7). Before testing, we thawed the samples (mean ± SD: 2 h 24 min ± 42 min).

The self-built setup made from acrylic glass (Fig. S1) was secured to the enclosure's grids, with additional wire mesh fitted in front of it. Opened glass vials containing the samples were inserted into two tubes accessible from the outside, so that the sample opening was positioned behind a mesh directly at the front opening facing the chimpanzee. The samples were spaced 56 cm apart from each other.

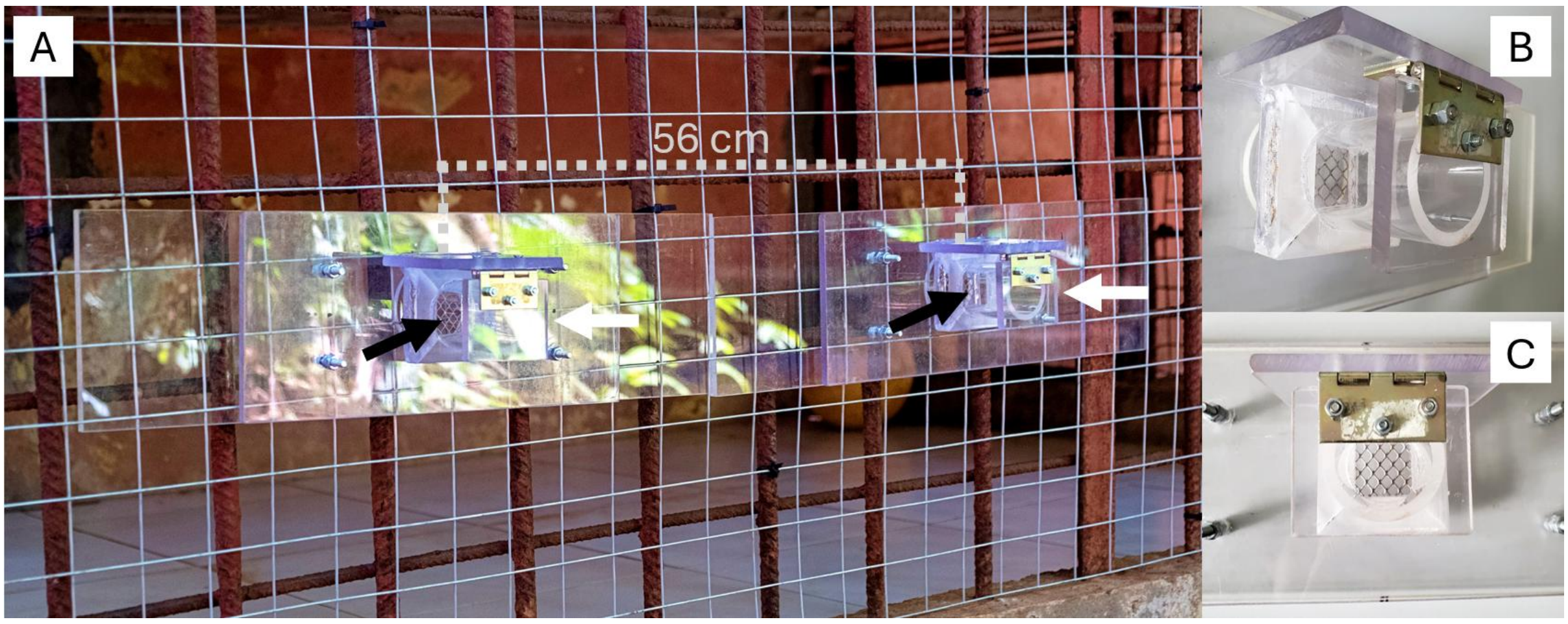

Fig. S1. Setup for bioassays. A: Original positioning during a trial, with two tubes (white arrows, 45 mm diameter, 56 cm apart from each other) for holding the opened glass vials with odour samples directly behind a mesh (black arrows). B: Lateral close-up view and C: frontal close-up view.

Samples were handled wearing gloves at all times and were left in the setup for five minutes from the moment the males were able to approach it. The behavioural tests were video recorded and afterwards analysed using the video recordings. For filming, we used at least one camera positioned directly in front of the setup, showing both samples simultaneously. In addition, depending on the enclosure characteristics, we filmed a side view from a diagonal front or diagonal rear angle. The person handling the samples and cameras (J.W.S.) was blind to the sample identity and kept out of sight and/or concealed any facial expressions by wearing sunglasses and a baseball cap during the tests.

The two samples within a single preference test always originated from different females to avoid the unnatural situation of encountering odours from the same female from two different time points. None of the males was presented with the same combination of female samples twice and at least four days of rest period were between tests involving the same male. Within each preference test, two samples from Dudley or two samples from Leipzig were presented, but never a mixture of the two locations. Due to the limited number of samples, odour samples were repeatedly used, but samples with the same number of uses were always paired together and the rate of investigations did not change between first usage and repeated usage trials. Usually, males were separated in an enclosure for the tests. For three groups a separation of single males was not possible and the setup was presented to a group of individuals. Males did not receive a reward for participating in the bioassays, so as not to induce learned behaviour.

Statistical Analysis

Additional explanation of statistical approach

If both predictors are included together (A), the estimated effect for each of them is investigated while controlling for the other predictor, i.e. we unravel the direct effect of one variable in addition to what the other predictor already explains. If only one predictor is included at a time (B), the effect of this predictor is estimated including the variance of the other variable, because it is not controlled for in the analysis. See (8,9) on mediating effects in causal inference.

Model parameters included in generalized linear mixed models

Variant A including *both* predictors, sexual swelling stage and menstrual cycle phase, in the same model to assess the direct effects for both. The random interactions of 'Swelling' and 'Cycle' within 'Compound' (indicated in bold) were the test predictors lacking the model for Likelihood ratio tests, first both at the same time for the overall test and then individually for assessing the separate effects.

Model parameters	Variables
Fixed effects:	Swelling, Cycle, Enclosure, GC–MS batch
Random effects:	Samples, Compound, Individual
Random interactions within Compound:	**Swelling, Cycle**, GC–MS batch
Random interactions within Individual:	Swelling, Cycle

Variant B including *only* menstrual cycle phase to assess its total effect. The random interaction of 'Cycle' within 'Compound' (indicated in bold) was the test predictor lacking the model for the Likelihood ratio test.

Model parameters	Variables
Fixed effects:	Cycle, Enclosure, GC–MS batch
Random effects:	Samples, Compound, Individual
Random interactions within Compound:	**Cycle**, GC–MS batch
Random interactions within Individual:	Cycle

Re-Analysis with reduced dataset

We have run the statistical analysis, including the generalized linear mixed models and permutational analyses of variances with both transformations, again with a reduced dataset. As the periovulatory phase (N = 8 samples) and also the luteal phase (N = 14) are represented by relatively fewer samples in the full dataset compared with the broader follicular time window (N = 52), this could reduce the likelihood of detecting effects. Therefore, in the reduced dataset, we used the same samples from the periovulatory (N = 8) and the luteal phase (N = 14) of the whole dataset, but only samples from a limited time window preceding the periovulatory window for the follicular phase (N = 25, including days -5 to -9 before last periovulatory day). This shortening caused the increasing swelling stage to be completely omitted in the reduced data set. Thus, the 47 samples of the reduced dataset only contain 29 sample from the maximal swelling, 6 from the decreasing phase and 12 from the flat phase.

RESULTS

Most affected compounds of the full dataset

Table S2. Compounds most affected by swelling stages and/or menstrual cycle phases with tentative compound identification (ID), average retention time (RT), similarity index (SI) indicating how well measured mass spectrum and reference spectrum matched, corresponding CAS number, molecular weight (MW) and compound class category. A numerical ranking of compounds affected by swelling stages (Top Swell) and menstrual cycle phases (Top Cyc) represent the strength of the effects (1 = strongest effect) and the differences between swelling stages and menstrual cycle phases are given in brackets (threshold of average + 2.5 SD: 0.58 for swelling stages and 0.27 for menstrual cycle phases). Some example references are given for studies on primates including humans that found the same compound. Known contaminants had been removed beforehand. Compounds with an asterisk (*) were confirmed using a reference standard.

**Compound ID**	**RT**	**SI**	**CAS**	**MW**	**Compound class**	**Top Swell**	**Top Cyc**	**References**
Acetic acid*	2.63	97	64-19-7	60	carboxylic acid	4 (0.66)		- Vaginal secretions of chimpanzees (10,11), humans (12), and rhesus macaques (13,14)<br>- Circumgenital gland secretions of female common marmosets (15)<br>- Sternal gland of mandrills (*Mandrillus sphinx*) (16)<br>- Faeces, urine, breath, skin, milk and saliva of humans (reviewed in 17))
Propanoic acid	3.67	92	79-09-4	74	carboxylic acid	1 (0.9)	3 (0.32)	- Vaginal secretions of chimpanzees (10,11), humans (12), and rhesus macaques (13,14)<br>- Circumgenital gland of female common marmosets (15)<br>- Faeces, urine, breath, skin, milk and saliva of humans (reviewed in 17)20/08/2026 12:35:00
Heptane, 2-methyl-	4.68	96	592-27-8	114	alkane	5 (0.63)		- Breath and saliva of humans (reviewed in 17)

Undecane	9.98	92	1120-21-4	156	alkane hydrocarbon	2 (0.66)		- Sternal scent gland of mandrills (16,18)<br>- Human axillary sweat (19), faeces, breath, skin, milk and saliva (reviewed in 17)
Methenamine*	14.95	97	100-97-0	140			1 (0.36)	- Breath of humans (reviewed in 17)<br>- Anogenital skin of common marmosets (5)
Cholesterol*	44.32	84	57-88-5	386	sterol	3 (0.66)	2 (0.35)	- Glandular secretions of ringtailed lemurs (20)

Results of the reduced dataset

The results of the analyses using the reduced dataset have fully confirmed the results of the initial analyses using the full dataset.

Chemical composition: When both variables were included (LRT full-null: $\chi2 = 49.96$, df = 2, $p < 0.001$), we found a direct effect of swelling stage (LRT swelling: $\chi2 = 42.90$, df = 1, $p < 0.001$), but not of menstrual cycle phase (LRT cycle: $\chi2 = 0$, df = 1, $p = 0.999$) on chemical compounds in the log-transformed model variant. When the response was normalised, neither swelling stage nor menstrual cycle phase had a significant effect (LRT full-null: $\chi2 = 3.71$, df = 2, $p = 0.156$). When we included only menstrual cycle phase, it significantly affected chemical composition (LRT: $\chi2 = 7.09$, df = 1, $p = 0.008$) with log-transformed, but not with normalised response (LRT: $\chi2 = 0$, df = 1, $p = 1$).

Chemical profiles: When both variables were included, swelling stage as well as menstrual cycle phase significantly affected whole chemical profiles with log-transformed (swelling: $R^2 = 0.13$, $F = 3.40$, $p = 0.001$ and cycle: $R^2 = 0.08$, $F = 2.03$, $p = 0.047$) and normalised response (swelling: $R^2 = 0.11$, $F = 2.68$, $p = 0.001$ and cycle: $R^2 = 0.09$, $F = 2.18$, $p = 0.010$). When only menstrual cycle phase was included, it affected the profiles neither with the log-transformed ($R^2 = 0.06$, $F = 1.39$, $p = 0.179$) nor with the normalised response ($R^2 = 0.07$, $F = 1.54$, $p = 0.062$).

REFERENCES

1. Daskalaki M, Drummer C, Behr R, Heistermann M. The use of alfaxalone for short-term anesthesia can confound serum progesterone measurements in the common marmoset: a case report. Primate Biol. 2022 Jul 27;9(2):23–8. doi:10.5194/pb-9-23-2022
2. Graham L, Schwarzenberger F, Möstl E, Galama W, Savage A. A versatile enzyme immunoassay for the determination of progestogens in feces and serum. Zoo Biol. 2001;20(3):227–36. doi:10.1002/zoo.1022
3. Struck M, Severud WJ, Chenaux-Ibrahim YM, J. Isaac E, Brown JL, Moore SA, et al. Refining the moose serum progesterone threshold to diagnose pregnancy. Conserv Physiol. 2023 Jan 1;11(1):coad003. doi:10.1093/conphys/coad003
4. Harlow CR, Sara G, Hodges JK, Hearn JP. The relationship between plasma progesterone and the timing of ovulation and early embryonic development in the marmoset monkey (*Callithrix jacchus*). J Zool. 1983 Oct;201(2):273–82. doi:10.1111/j.1469-7998.1983.tb04276.x
5. Kücklich M, Weiß BM, Birkemeyer C, Einspanier A, Widdig A. Chemical cues of female fertility states in a non-human primate. Sci Rep. 2019;9(1):1–12. doi:10.1038/s41598-019-50063-w PubMed PMID: 31548568.
6. Stein SE. An integrated method for spectrum extraction and compound identification from gas chromatography/mass spectrometry data. J Am Soc Mass Spectrom. 1999 Aug 1;10(8):770–81. doi:10.1016/S1044-0305(99)00047-1
7. Lenochova P, Roberts SC, Havlicek J. Methods of human body odor sampling: the effect of freezing. Chem Senses. 2009 Feb 1;34(2):127–38. doi:10.1093/chemse/bjn067
8. Franks DW, Ruxton GD, Sherratt T. Ecology needs a causal overhaul. Biol Rev. 2025 Oct;100(5):1950–69. doi:10.1111/brv.70029
9. Pearl J. The causal mediation formula—a guide to the assessment of pathways and mechanisms. Prev Sci. 2012 Aug 1;13(4):426–36. doi:10.1007/s11121-011-0270-1
10. Fox GJ. Potentials for pheromones in chimpanzee vaginal fatty acids. Folia Primatol (Basel). 1982 Feb 8;37(3–4):255–66. doi:10.1159/000156036
11. Matsumoto-Oda A, Oda R, Hayashi Y, Murakami H, Maeda N, Kumazaki K, et al. Vaginal fatty acids produced by chimpanzees during menstrual cycles. Folia Primatol (Basel). 2003 Feb 8;74(2):75–9. doi:10.1159/000070000
12. Michael RP, Bonsall RW, Kutner M. Volatile fatty acids, “copulins”, in human vaginal secretions. Psychoneuroendocrinology. 1975;1(2):153–63. doi:10.1016/0306-4530(75)90007-4
13. Curtis RF, Ballantine JA, Keverne EB, Bonsall RW, Michael RP. Identification of primate sexual pheromones and the properties of synthetic attractants. Nature. 1971;232:396–8.
14. Michael RP, Keverne EB, Bonsall RW. Pheromones: Isolation of male sex attractants from a female primate. Science. 1971 May 28;172(3986):964–6. doi:10.1126/science.172.3986.964
15. Smith. Female marmoset monkeys (*Callithrix jacchus*) can be identified from the chemical composition of their scent marks. Chem Senses. 2001 Jun 1;26(5):449–58. doi:10.1093/chemse/26.5.449

16. Vaglio S, Minicozzi P, Romoli R, Boscaro F, Pieraccini G, Moneti G, et al. Sternal gland scent-marking signals sex, age, rank, and group identity in captive mandrills. Chem Senses. 2015 Dec 26;00:1–10. doi:10.1093/chemse/bjv077
17. Costello B de L, Amann A, Al-Kateb H, Flynn C, Filipiak W, Khalid T, et al. A review of the volatiles from the healthy human body. J Breath Res. 2014 Jan 13;8(1). doi:10.1088/1752-7155/8/1/014001
18. Setchell JM, Vaglio S, Moggi-Cecchi J, Boscaro F, Calamai L, Knapp LA. Chemical composition of scent-gland secretions in an Old World monkey (*Mandrillus sphinx*): Influence of sex, male status, and individual identity. Chem Senses. 2010 Mar 1;35(3):205–20. doi:10.1093/chemse/bjp105
19. Curran AM, Rabin SI, Furton KG. Analysis of the uniqueness and persistence of human scent. Forensic Sci Commun [Internet]. 2005 Apr 1 [cited 2026 Apr 1];7(2). Available from: https://go.gale.com/ps/i.do?p=AONE&sw=w&issn=15288005&v=2.1&it=r&id=GALE%7CA139298569&sid=googleScholar&linkaccess=abs
20. Scordato ES, Dubay G, Drea CM. Chemical composition of scent marks in the ringtailed lemur (Lemur catta): Glandular differences, seasonal variation, and individual signatures. Chem Senses. 2007;32(5):493–504. doi:10.1093/chemse/bjm018 PubMed PMID: 17488747.
